# In Vivo Dedifferentiation of Adult Adipose Cells

**DOI:** 10.1371/journal.pone.0125254

**Published:** 2015-04-22

**Authors:** Yunjun Liao, Zhaowei Zeng, Feng Lu, Ziqing Dong, Qiang Chang, Jianhua Gao

**Affiliations:** Department of Plastic and Cosmetic Surgery, Nanfang Hospital, Southern Medical University, Guang Zhou, Guang Dong, P.R, China; Universidad Pablo de Olavide, Centro Andaluz de Biología del Desarrollo-CSIC, SPAIN

## Abstract

**Introduction:**

Adipocytes can dedifferentiate into fibroblast-like cells in vitro and thereby acquire proliferation and multipotent capacities to participate in the repair of various organs and tissues. Whether dedifferentiation occurs under physiological or pathological conditions in vivo is unknown.

**Methods:**

A tissue expander was placed under the inguinal fat pads of rats and gradually expanded by injection of water. Samples were collected at various time points, and morphological, histological, cytological, ultrastructural, and gene expression analyses were conducted. In a separate experiment, purified green fluorescent protein+ adipocytes were transplanted into C57 mice and collected at various time points. The transplanted adipocytes were assessed by bioluminescence imaging and whole-mount staining.

**Results:**

The expanded fat pad was obviously thinner than the untreated fat pad on the opposite side. It was also tougher in texture and with more blood vessels attached. Hematoxylin and eosin staining and transmission electron microscopy indicated there were fewer monolocular adipocytes in the expanded fat pad and the morphology of these cells was altered, most notably their lipid content was discarded. Immunohistochemistry showed that the expanded fat pad contained an increased number of proliferative cells, which may have been derived from adipocytes. Following removal of the tissue expander, many small adipocytes were observed. Bioluminescence imaging suggested that some adipocytes survived when transplanted into an ischemic-hypoxic environment. Whole-mount staining revealed that surviving adipocytes underwent a process similar to adipocyte dedifferentiation in vitro. Monolocular adipocytes became multilocular adipocytes and then fibroblast-like cells.

**Conclusions:**

Mature adipocytes may be able to dedifferentiate in vivo, and this may be an adipose tissue self-repair mechanism. The capacity of adipocytes to dedifferentiate into stem cell-like cells may also have a more general role in the regeneration of many tissues, notably in fat grafting.

## Introduction

Adipose tissue is important for energy storage and is the largest endocrine organ in the body [1]. It contains numerous cell types, including adipocytes, adipose-derived stromal cells (ASCs), endothelial cells, mural cells, fibroblasts, and blood cells [2]. Adipocytes are the prominent cell type within adipose tissue and are responsible for the main function of this tissue, namely, lipid metabolism [3]. Adipocytes are generally considered to be at the terminal stage of differentiation, and having lost their proliferative ability, are stationary. However, recent data suggest that mature adipocytes can reversibly change their phenotype and transform into cells with a different morphology and physiology via a process termed transdifferentiation [4,5]. It is unclear whether this process directly converts one cell type into another or involves stepwise dedifferentiation of the primary cell into an intermediate cell type that can differentiate into a new lineage. Also, the dedifferention of mature adipocytes in vivo is not well studied.

Dedifferentiation involves a terminally differentiated cell reverting into a less differentiated stage within its own lineage. Most dedifferentiation events occur infrequently. However, mammalian somatic cells, such as Schwann cells, cardiac myocytes and germ cells, can dedifferentiate in response to stress [6,7,8]. In stress conditions, dedifferentiation may be one way by which cells react to minimize damage [9]. Mature adipocytes can discard lipid droplets and dedifferentiate into fbroblast-like cells in vitro via a technique called ceiling culture [10]. Under appropriate culture conditions, dedifferentiated fat (DFAT) cells can not only redifferentiate into adipocytes but also into osteoblasts, chondrocytes, smooth muscle cells, cardiomyocytes, vascular endothelial cells and neural cells [11,12]. DFAT cells also contribute to sphincter function [13], participate in infarcted cardiac tissue repair [14], and even function in the central nervous system [15]. In addition, the transcriptional signature of DFAT cells is characterized by significant decreases in functional phenotype-related genes and increases in genes related to cell proliferation, altered cell morphology, and regulation of differentiation [16].

Based on these studies, we hypothesized that mature adipocytes can dedifferentiate into lipid droplet-free DFAT cells in vivo under extreme conditions, and that DFAT cells may redifferentiate into mature adipocytes under specific environmental conditions. To test these hypotheses, we placed a tissue expander under the inguinal fat pads of rats to increase mechanical pressure within the local tissue, after which the tissue expander was removed. Histological, PCR, transmission electron microscopy (TEM), and immunohistochemistry analyses were performed to examine the dedifferentiation and redifferentiation of adipocytes. Moreover, another animal model was used to further examine the dedifferentiation process in vivo. Mature adipocytes isolated from green fluorescent protein (GFP) transgenic mice were traced following transplantation into the fat pads of C57 mice.

## Materials and Methods

### Ethics Statement

The animal experimental protocols were approved by the Southern Medical University Laboratory Animal Administration Committee and the experiment was performed according to the Southern Medical University Guidelines for Animal Experimentation. All efforts were made to minimize suffering.

### Establishment of animal models

Two animal models were used: (1) a rat model in which a tissue expander was implanted, and (2) a GFP+ adipocyte transplantation mouse model to track cells during dedifferentiation. All animal experiments were performed according to the Guide for the Care and Use of Laboratory Animals (NIH Publication no. 85–23, revised 1996). All animals were obtained from the Experimental Animal Center of Southern Medical University.

A tissue expander was implanted [17]. Twenty-five male Sprague-Dawley rats weighing 250–300 g were used. Rats were anesthetized with chloral hydrate (100 mg/kg intraperitoneally) and the abdominal skin was opened by a midline incision. Both fat pads were exposed and a 20-ml cylindrical expander (Wanhe, Canton, China) was placed under the left inguinal fat pad. Gentamicin was given intraoperatively. On postoperative Day 10, the wound had completely closed and expansion of the tissue expander was commenced by injecting 3 ml daily up to 42 ml. The expanded inguinal and contralateral fat pads tissue were harvested on Day 4, 7, 10, or 14 after the first saline injection (n = 5 per time point). The day after saline injections were completed, the expander was removed (n = 5). Fourteen days later, the remaining fat pads were harvested. All fat pads were examined as described below.

For transplantation of isolated GFP+ adipocytes, 8-week-old male GFP transgenic mice were used as donors (n = 5) and 8-week-old male C57 mice were used as recipients (n = 18). Suspensions of mature adipocytes were obtained from the epididymal fat pads of GFP transgenic mice using a previously described technique [5]. Fragments of adipose tissue were incubated for 15 min at room temperature in phosphate-buffered saline (PBS) containing 3% human albumin (Sigma-Genosys, Cambridge, UK). After centrifugation at 150 g for 7 min to remove blood cells and debris, floating fat lobules were collected from the supernatant, minced, transferred to 50-ml tubes, and digested at 37°C for 1.5 h with PBS containing 2 mg/ml type I collagenase (Invitrogen, Carlsbad, CA). Centrifugation at 150 g for 7 min yielded a pure fraction of isolated, floating adipocytes and a pellet containing the stromal-vascular fraction. Aliquots of mature isolated adipocytes suspended in PBS were immediately used for injection. Samples of mature isolated adipocytes were also cultured and processed for morphological analyses. Multiple (3–4) injections of donor mature isolated adipocytes were made into the right fat pads of recipient mice. The injection volume was 50 μL (30,000 cells per experiment).

### Histological examination

For hematoxylin and eosin (H&E) staining and immunohistochemistry assays, fat pads were fixed with 4% paraformaldehyde for 24 h and embedded in paraffin. Sections (4 μm thick) were stained with H&E for conventional morphological evaluation, while the mouse anti-rat Ki67 antibody (Dako, Carpinteria, CA, USA) was used to detect proliferating cells. All samples were assessed under an OLYMPUS IX71 microscope and photographed using OLYMPUS DP controller software.

To determine the source of Ki67+ cells, harvested adipose tissue was zinc-fixed (Zinc Fixative; BD Biosciences, San Diego, CA, USA), paraffin-embedded, sectioned into 6 μm-thick slices, and immunostained using the following primary antibodies: rabbit anti-Ki67 (Thermo Fisher Scientific, Fremont, CA, USA) and guinea-pig anti-perilipin (PROGEN, Heidelberg, Germany). To further identify the blood vessels within the sample were stained with Lectin GS-II Alexa Fluor 594 Conjugate (Thermo Fisher Scientific, MA, USA). For double fluorescence staining, Alexa Fluor 488 and Alexa Fluor 568-conjugated secondary antibodies (Molecular Probes) appropriate for each primary antibody were used. Nuclei were stained with Hoechst 33342 (Sigma, Missouri, IL, USA). Samples were washed and observed directly by confocal microscopy (Leica TCS SP2; Leica Microsystems GmbH, Wetzlar, Germany).

### Electron microscopy

Fragments of fat tissue were fixed in 0.1 M phosphate buffer (pH 7.4) containing 2% glutaraldehyde and 2% formaldehyde, processed for electron microscopy [18], and examined using a CM10 Philips transmission electron microscope (TEM) (Philips, Eindhoven, The Netherlands).

### Real-time PCR

All rats were anesthetized with chloral hydrate (100 mg/kg intraperitoneally). The fat tissue was quickly excised, immediately frozen in liquid nitrogen, and stored at −80°C. Total RNA was extracted from 50 mg of frozen tissue using the RNeasy Lipid Tissue Mini Kit (Qiagen, Hilden, Germany) according to the manufacturer’s instructions. cDNA was amplified in 40 cycles using a QuantiTect Reverse Transcription Kit (Qiagen, Hilden, Germany) and a Rotor-Gene 3000 Real-Time PCR Detection System (CorbettResearch, Sydney, Australia). GAPDH was used as the reference gene against which expression of Peroxisome proliferator-activated receptor-γ (PPARγ), adiponectin and CCAAT-enhancer-binding protein α (C/EBPα) were normalized. Expression levels were calculated by the 2-ΔΔCt method [19]. The primer sequences were as follows: PPARγ, sense 5´-GCGGAGATCTCCAGTGATATC-3´ and antisense 5´-TCAGCGACTGGGACTTTTCT-3´; adiponectinsense, 5´-AAAGGAGAGCCTGGAGAAGC-3´ and antisense 5´-AAAGGAGAGCCTGGAGAAGC-3´; CCAAT-enhancer-binding protein α (C/EBPα), 5´-CAAAGCCAAGAAGTCGGTGGACAA-3´, 5´-TCATTGTGACTGGTCAACTCCAGC-3´; GAPDH, sense 5´-AACTTTGGCATTGTGGAAGG-3´ and antisense 5´-CCCTGTTGCTGTAGCCGTAT-3´.

### Culture and identification of GFP+ DFAT cells in vitro

A portion of floating primary cells were fixed in 4% formaldehyde (Wako) for 10 min, washed with PBS, and stained with Hoechst 33342 (Sigma, Missouri, IL, USA) for 30 min. The remainder of the mature adipocytes were placed in culture flasks (BD Falcon 3107, Bedford, MA) containing Dulbecco’s modified Eagle’s medium (Nissui Pharmaceutical, Tokyo, Japan) supplemented with 10% fetal bovine serum (Moregate Biotech, Queensland, Australia). The flasks were completely filled with medium to create an air-free environment, inverted, and incubated in a humidified atmosphere of 5% CO_2_. The cells floated up through the medium and adhered to the top (ceiling) of the flask. Approximately 10 days later, the cells were firmly adhered and had a fibroblast-like morphology with no visible fat droplets. Cells were observed using a fluorescence microscope (Olympus IX71, Tokyo, Japan). The brightness and contrast of the images were adjusted using Photoshop 6 software (Adobe Systems; Mountain View, CA, USA).

### Differentiation of GFP+ DFAT cells

*In vitro* multi-lineage differentiation of dedifferentiated adipocytes was induced in the control medium supplemented with one of the three formulas described below. *In vitro*-cultured dedifferentiated adipocytes were detected using Oil-red O, Alizarin red, and Alcian blue staining, which identified fat, bone, and cartilage cells, respectively, differentiated from dedifferentiated adipocytes.

### In vivo bioluminescence imaging

After injecting mice with GFP+ adipocytes, each mouse was anesthetized with isoflurane and in vivo bioluminescence imaging was performed using the Xenogen IVIS-Spectrum Imaging System (Xenogen; Caliper Life Sciences, Inc.). Imaging was performed 1, 2, 4, 7, 10, and 21 days after injection.

### Whole-mount staining

Fat pad samples were obtained from C57 mice at 1, 2, 4, 7, 10, and 21 days after injection. Whole-mount staining of fresh adipose tissue was performed as described previously [20]. Briefly, adipose tissue was cut into 0.5–1 mm thick pieces and incubated with an anti-perilipin antibody (Santa Cruz Biotechnology, Santa Cruz, CA) at 4°C overnight. Thereafter, samples were incubated with Alexa Fluor 547-conjugated AffiniPure donkey anti-rabbit IgG (H+L) for 2 hrs, washed 3 times, incubated with Hoechst 33342 (Sigma, Missouri, IL, USA) for 30 min to stain nuclei, and then washed again. Samples were observed directly by confocal microscopy (Leica TCS SP2; Leica Microsystems GmbH, Wetzlar, Germany).

### Statistical analysis

Data are expressed as the mean and standard error of the mean. For real-time PCR analyses, asterisks represent statistical significance as P<0.05. Welch’s t test was performed using SPSS 13.0 (SPSS Inc., Chicago, IL).

## Results

### General and histological observations of adipose tissue after expansion

**Fig 1:**
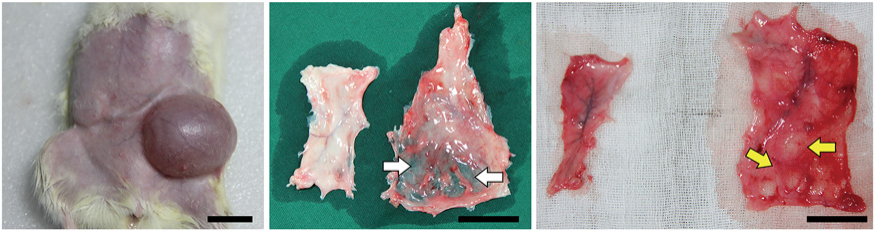
Rat fat pad expantion. Left: Rats after transplantation of the expander. Middle: The expanded fat pad became thinner (white arrows). Right: After removal of the expander, the regrowth of adipose-liked tissue (yellow arrows) was seen. Scale bar = 2 cm.

The expander was well tolerated by all animals. Necrosis of the skin and soft tissue was not observed (Fig 1, left). After carefully separating the fat pad from the skin, the axial blood vessel of the fat pad was seen. The expanded fat pad was obviously thinner than the non-treated fat pad on the opposite side (white arrow) (Fig 1, middle). It was also firmer in texture and darker in color, with more blood vessels attached. Two weeks after the tissue expander was removed, the expanded fat pad had softened and regained the color as the non-treated fat pad on the opposite side. Moreover, there was white adipose-like tissue in the expanded region (yellow arrows) (Fig 1, right).

**Fig 2:**
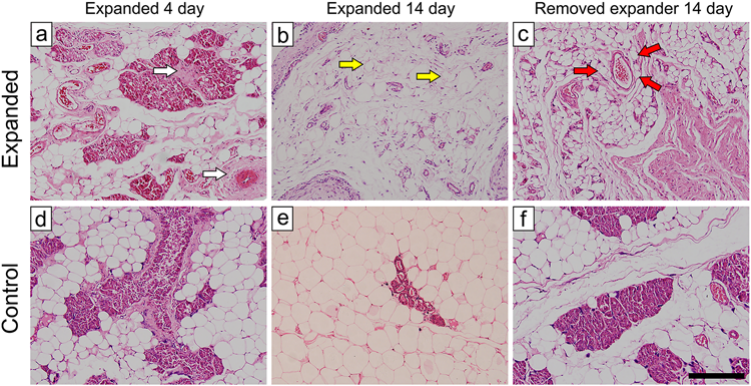
Histology observation of the fat pad. Normal adipocytes were uniform in size and had a normal morphology. During expansion, the growth of connect tissue (white arrow) and the reduced volume of adipocytes (yellow arrow) were observed. After removing the expander, many adipocytes of small sizes and a normal morphology emerged around the new vessels (red arrow). Scale bar = 200 μm.

The histological structure of expanded adipose tissue was different from that of non-treated adipose tissue. In non-treated adipose tissue, adipocytes were similar in size and had a normal morphology. After 4 days of expansion, the adipose tissue structure was disordered and a small amount of fibrous tissue had formed (white arrow) (Fig 2a and 2d). Adipocytes had a normal morphology and their number had not obviously decreased. There was a loose and thin capsule where the fat pad contacted the tissue expander. After 7 days of expansion, the number of adipocytes had decreased and the number of interstitial cells had increased. Tissue fibrosis was aggravated, the external capsule had thickened, and there were newly formed blood vessels in adipose tissue and the capsule. After 10 days of expansion, the number of adipocytes had further decreased, normal adipocytes were almost completely absent from the capsule region, and the external envelope was compact. After 14 days of expansion, only a small number of adipocytes with a normal morphology were observed in the expanded adipose tissue (yellow arrow) (Fig 2b and 2e), and there were large numbers of fibroblast-like cells and new vessels. Two weeks after removing the expander, tissue fibrosis was alleviated, but the capsule remained. Many small adipocytes of a normal morphology (red arrow) emerged around the new vessels (Fig 2c and 2f).

### Immunohistochemical analysis of the expanded adipose tissue

**Fig 3:**
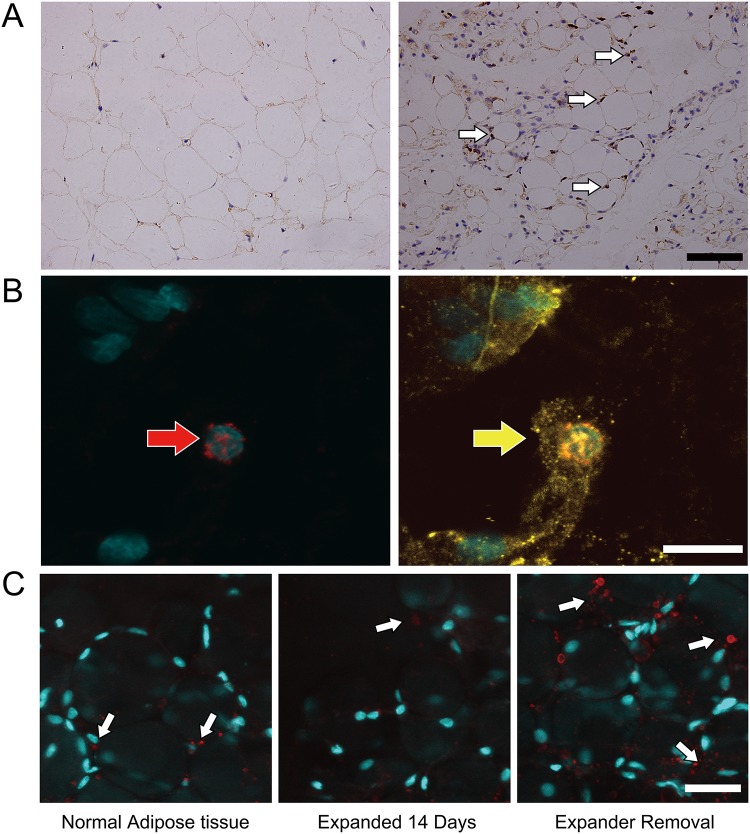
The proliferation and angiogenesis of the fat pad. A: The number of Ki67+ cells (white arrow) was increased during expension (day 0 left, day 14 right). Scale bar = 50 μm. B: Some of the Ki67+ cells (red arrow) were also perilipin+ (yellow arrow). Scale bar = 10 μm. C: The number of blood vessels (white arrow) did not decrease significantly, and when removing the expander, the angiogenic level increased. Scale bar = 25 μm.

Expression of Ki67 suggests that cells are in a proliferative state. The number of Ki67+ cells (white arrow) was increased in expanded adipose tissue (Fig 3A, day 0 left, day 14 right). Most of the Ki67+ cells were seen in the connect tissue. Immunofluorescence confirmed the presence of cells positive for both Ki67 (red arrow) and perilipin (yellow arrow) (Fig 3B), which were likely proliferating mature DFAT cells or adipose-derived stem/progenitor cells. In addition, after the implantation of the expander, the number of blood vessel (white arrow) did not increase. However, when removing the expander, the angiogenic level increased (Fig 3C).

### Emergence of adipocytes containing multilocular lipid droplets

**Fig 4:**
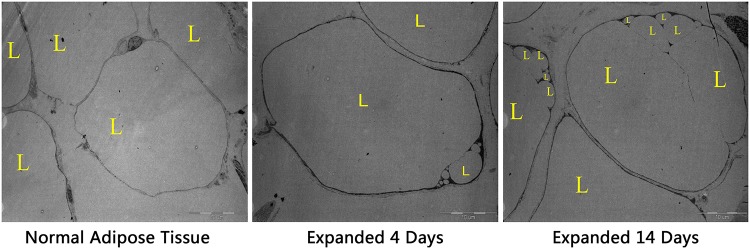
TEM analysis of the expanded tissue. During the expansion, the number of adipocytes with monolocular lipid droplets increased over time. L, lipid droplet.

During dedifferentiation, lipids are divided into daughter cells symmetrically or asymmetrically. Alternatively, lipids are directly expelled into the medium prior to cell division. TEM was used to determine whether adipocytes in expanded fat pads contained multilocular lipid droplets. Adipocytes in non-treated adipose tissue were closely connected. The nucleus was positioned to one side of the adipocyte and the majority of the intracellular space was occupied by an extremely large lipid droplet. In expanded adipose tissue, the intercellular space was larger, there was a large amount of fibrillar components around adipocytes, and there were bilocular and multilocular lipid droplets in adipocytes. In addition, the number of multilocular adipocytes increased during the expansion. This suggests that adipocytes discard their lipid droplets and undergo dedifferentiation upon tissue expansion (Fig 4).

### Adipogenic gene expression

**Fig 5:**
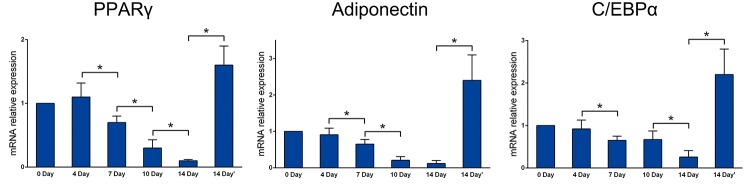
The expression level of adipogenic gene. During expansion, PPARγ, adiponectin and C/EBPα expression levels decreased gradually. The expression level of them increased significantly after expander removel (*P<0.05).

PPARγ expression in expanded tissue obviously decreased over time. Two weeks after removing the expander, PPARγ expression increased and even exceeded the normal level. Furthermore, we also analysis the expression levels of adiponectin and CCAAT-enhancer-binding protein α (C/EBPα) by PCR. The result suggested that the expression levels changing trend of the both adiponectin and C/EBPα were similar to PPARγ. The expression dropped over time after expander implanted and increased after removing the pressure (Fig 5).

### Purification and ceiling adherent culture of adipocytes and the differentiation potential of DFAT cells

**Fig 6:**
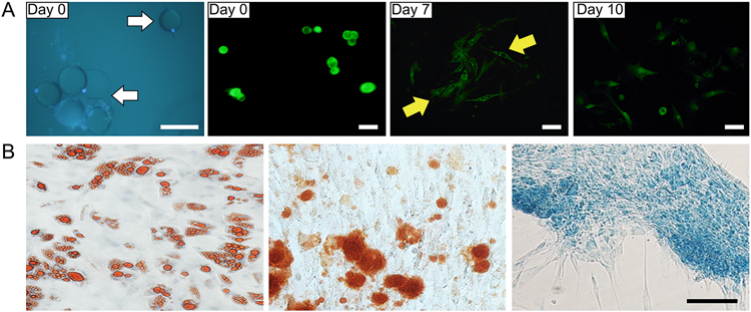
Morphology change of GFP+ adipocytes during dedifferention. A: monolocular mature adipocytes (white arrow) with GFP signal at day 0. After 7 days ceiling culture, cells with multilocular lipid droplets were observed (yellow arrow). 10 days later, the cells had a fibroblast-like morphology with no visible fat droplets. Scale bar = 100 μm. B: The adipogenic (left), osteogenic (middle) and chondrogenic (right) differentiation of DFAT cells. Scale bar = 25 μm.

Nuclear staining of the cell suspension that was isolated from GFP transgenic mice was conducted to determine the purity of adipocytes. All adipocytes were mononuclear (white arrow). After adherent culture, these mature adipocytes became spindle cell pattern and with multilocular lipid droplets (yellow arrow). 10 days later, the cells were firmly adhered and had a fibroblast-like morphology with no visible fat droplets (Fig 6A). The differentiation potential of DFAT cells was analyzed by culturing the cells under conditions favorable for adipogenic, osteogenic and chondrogenic differentiation. DFAT cells were incubated in media known to induce an adipogenic, osteogenic, or chondrogenic lineage. Adipogenic differentiation was determined by Oil Red O staining of intracellular lipid droplets (Fig 6B, left), osteogenic differentiation through Alizarin red S staining of matrix mineralization (Fig 6B, middle), and chondrogenic differentiation through Alcian blue staining of cartilage-specific proteoglycans (Fig 6B, right).

### Viability and morphological changes of adipocytes after transplantation

**Fig 7:**
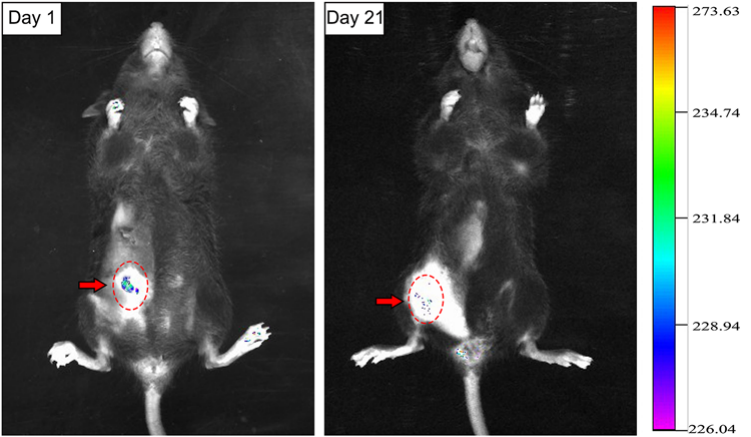
The retention of GFP+ adipocytes after transplantation. On Day 1 after transplantation, there was intense green fluorescent protein fluorescence (red arrow) in the injected region. On Day 21 after transplantation, there were only a few scattered regions of fluorescence.

The survival of GFP+ adipocytes at various time points after transplantation was monitored by bioluminescence imaging. On Day 1 after transplantation, there was intense green fluorescence in the region where cells were injected (red dotted line circle). During Day 2–4 after transplantation, the fluorescence intensity decreased, which suggests that adipocytes had begun to undergo apoptosis. During Days 7–10 after transplantation, the fluorescence intensity further decreased and fluorescence almost completely disappeared. On Day 21 after transplantation, the fluorescence intensity was similar to that observed on Day 10 and there were very few GFP+ regions, suggesting that very few transplanted adipocytes survived the ischemic-hypoxic period (Fig 7).

**Fig 8:**
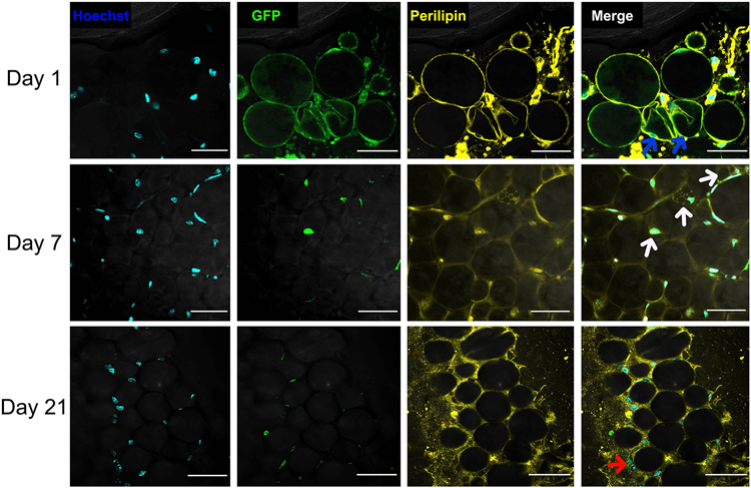
Fate of GFP+ adipocytes after transplantation. On Day 1 after transplantation, some GFP+ adipocytes exhibited membrane contraction (blue arrow). On Day 7, some surviving GFP+ adipocytes had multilocular lipid droplets (white arrow). On Day 21, very few adipocytes survived and those had completely discarded their lipid droplets and changed into fibroblast-like cells (red arrow). Scale bar = 100 μm.

Whole-mount staining revealed that following transplantation, only a few adipocytes survived, the morphology of which was similar to that of dedifferentiated adipocytes in vitro. On Day 1 after transplantation, most transplanted adipocytes were alive; however, some had contracted membranes (blue arrow). On Day 7 after transplantation, there were GFP+ multilocular adipocytes (white arrow) in adipose tissue, suggesting their lipid droplets had been discarded. On Day 21 after transplantation, there were GFP+ fibroblast-like cells (red arrow) in the mesenchyme of adipose tissue. We believed that this is the final morphology of transplanted mature adipocytes after dedifferentiation in vivo (Fig 8).

## Discussion

Mammals have a limited capacity to regenerate and restore their tissues and organs. This can be achieved by activating somatic stem cells located in a niche or by inducing differentiated cells to proliferate [21]. As a dynamic organ, approximately 10% of adipocytes in adipose tissue renew annually in adults [22]. It is traditionally thought that as progenitors of adipose tissue, ASCs play the most important role in all types of adipose tissue remodeling, including developmental growth, hyperplasia in obesity, post-injury repair, and ischemic [23]. Each adipocyte is directly attached to a capillary, which is easily damaged by injury or hypoxia [24]. However, it has been proposed that dedifferentiation of mature adipocytes and their progeny is an important source of regenerated adipocytes [25]. The dedifferentiation of adipocytes has been observed in many in vitro experiments; however, it remains unknown whether adipocytes dedifferentiate in vivo. Our data indicate that upon tissue expansion, mature, round, lipid-laden adipocytes were converted into multilocular adipocytes or lipid-free spindly fibroblasts. Meanwhile, expression of PPARγ, the master adipogenic regulator, was significantly reduced. During this process, adipocytes acquired the ability to proliferate and redifferentiate, as occurs during dedifferentiation.

We created a stress condition for adipose tissue by inducing tissue expansion. In this environment, the number of mature adipocytes was decreased. PPARγ is mainly expressed by adipocytes in adipose tissue and is one of the most important regulatory genes in the differentiation of adipocytes [26]. The decreased expression of PPARγ suggests that adipocytes in the expanded adipose tissue gradually decreased, which is consistent with our histological observations. Electron microscopy of expanded adipose tissue suggested that adipocytes may discard their lipid droplets and then occur dedifferentiation. However, an important question was left unanswered. During adipogenesis, ASCs are converted from spindly fibroblasts into multilocular immature adipocytes and terminal, round, lipid-laden, mature adipocytes [27]. Therefore, from their morphology alone, we could not determine whether the fibroblast-like cells observed were newly formed or undergoing dedifferentiation. It has been suggested that an apoptotic cell activates stem cells and thereby stimulates generation of a new cell to replace the dying cell; this process is called “compensatory proliferation” [28]. Adipocytes die easily under ischemic conditions, whereas ASCs are activated and contribute to adipose tissue repair [29]. To rule out the possibility that the fibroblast-like cells observed were actually derived from ASCs and to further determine the differentiation state of adipocytes in this stress condition, we tracked transplanted GFP+ adipocytes in mice to examine how they changed. Microscopic analyses showed that our preparations from GFP transgenic mice only contained adipocytes and were not contaminated by any other cells. Bioluminescence imaging showed that adipocytes could survive in this stress condition. But only a few monolocular adipocytes changed into multilocular adipocytes and then into fibroblast-like cells, consistent with our in vitro experiment. The data confirmed that fibroblast-like cells were derived from adipocytes, not from stem cells. In other words, mature adipocytes dedifferentiated in vivo under stress condition.

There are several examples of dedifferentiation in non-mammalian vertebrates [30,31]. By contrast, the capacity for regeneration via dedifferentiation is limited in mammals [32]. However, in the current study, mature adipocytes dedifferentiated towards proliferating immature cells in a rodent model. Such processes may help to combat extreme stress conditions that endanger the stem cell pool within adipose tissue. Generally, ASCs are thought to safeguard adipose tissue homeostasis by replenishing depleted cells, particularly under conditions of severe stress (such as non-vascularized grafting) [33]. Regeneration of adipose tissue following removal of the tissue expander involved the emergence of small adipocytes and a significant increase in PPARγ expression. Normally, regeneration of adipose tissue involves ASCs; however, we speculate that mature adipocytes also participate in this process by dedifferentiation.

In addition to adipose tissue, many organs and tissues in the mammals contain adipocytes, including bone marrow, thymus, parotid glands, parathyroid glands, pancreas, heart, and skeletal muscle. The roles of adipocytes in these regions are largely unknown, but adipocytes can highly express embryonic stem cell markers, such as Oct4, Sox2, c-Myc, and Nanog, after dedifferentiating [34]. Thus, they may represent a reservoir of pluripotent cells in dynamic equilibrium with organ-specific cellular components and be capable of phenotypic transformation. For example, transdifferentiation of adipogenic-differentiated cells into osteogenic- or chondrogenic-differentiated cells occurs via dedifferentiation [35].

The mechanism by which dedifferentiation occurs remains obscure. One theory is that the dedifferentiation of mature cells involves their relocation into a niche that harbors stem cell environmental cues, fusion of these cells with cells of a greater potency that have a dominant effect, specific gene overexpression, and epigenetic modulation [9]. Our study only observed the in vivo dedifferentiation of adipocytes, but did not further explore the underlying mechanisms. Mechanical forces might influence the proliferation and differentiation of cells [36]. It is unclear whether in vivo dedifferentiation is caused by the hypoxic-ischemic microenvironment and/or external mechanical force. Dedifferentiated cells have a hypo-methylated status, a possible characteristic of cancer cells [37]; therefore, dedifferentiation is often associated with tumorigenesis. Consequently, we must ensure that adipocytes can dedifferentiate safely in vivo.

Adipose tissue is considered an ideal soft-tissue filler in plastic and cosmetic surgery. However, the outcome of fat grafting is variable due to the hypoxic-ischemic microenvironment [38]. Preadipocytes consume significantly less oxygen than mature adipocytes [39]. Dedifferentiation of adipocytes and changing the vulnerability of adipocytes prior to grafting may improve such transplantation. Identification of the molecular mechanisms underlying dedifferentiation of adipocytes could offer new targets for future pharmacological interventions in the fields of regenerative medicine and tissue engineering.

## References

[1] McGown C, Birerdinc A, Younossi ZM. Adipose tissue as an endocrine organ. Clin Liver Dis. 2014; 18: 41–58. 10.1016/j.cld.2013.09.012 24274864

[2] Eto H, Suga H, Matsumoto D, Inoue K, Aoi N, Kato H, et al Characterization of structure and cellular components of aspirated and excised adipose tissue. Plast Reconstr Surg. 2009; 124: 1087–1097. 10.1097/PRS.0b013e3181b5a3f1 19935292

[3] Hausman GJ, Dodson MV. Stromal Vascular Cells and Adipogenesis: Cells within Adipose Depots Regulate Adipogenesis. J Genomics. 2013; 1: 56–66. 10.7150/jgen.3813 25031656 PMC4091429

[4] Rosenwald M, Perdikari A, Rulicke T, Wolfrum C. Bi-directional interconversion of brite and white adipocytes. Nat Cell Biol. 2013; 15: 659–667. 10.1038/ncb2740 23624403

[5] De Matteis R, Zingaretti MC, Murano I, Vitali A, Frontini A, Giannulis I, et al In vivo physiological transdifferentiation of adult adipose cells. Stem Cells. 2009; 27: 2761–2768. 10.1002/stem.197 19688834

[6] Chen ZL, Yu WM, Strickland S. Peripheral regeneration. Annu Rev Neurosci. 2007; 30: 209–233. 17341159 10.1146/annurev.neuro.30.051606.094337

[7] Bersell K, Arab S, Haring B, Kuhn B. Neuregulin1/ErbB4 signaling induces cardiomyocyte proliferation and repair of heart injury. Cell. 2009; 138: 257–270. 10.1016/j.cell.2009.04.060 19632177

[8] Barroca V, Lassalle B, Coureuil M, Louis JP, Le Page F, Testart J, et al Mouse differentiating spermatogonia can generate germinal stem cells in vivo. Nat Cell Biol. 2009; 11: 190–196. 10.1038/ncb1826 19098901

[9] Shoshani O, Zipori D. Mammalian cell dedifferentiation as a possible outcome of stress. Stem Cell Rev. 2011; 7: 488–493. 10.1007/s12015-011-9231-0 21279479

[10] Sugihara H, Yonemitsu N, Miyabara S, Yun K. Primary cultures of unilocular fat cells: characteristics of growth in vitro and changes in differentiation properties. Differentiation. 1986; 31: 42–49. 3732657 10.1111/j.1432-0436.1986.tb00381.x

[11] Jumabay M, Abdmaulen R, Urs S, Heydarkhan-Hagvall S, Chazenbalk GD, Jordan MC, et al Endothelial differentiation in multipotent cells derived from mouse and human white mature adipocytes. J Mol Cell Cardiol. 2012; 53: 790–800. 10.1016/j.yjmcc.2012.09.005 22999861 PMC3523675

[12] Poloni A, Maurizi G, Leoni P, Serrani F, Mancini S, Frontini A, et al Human dedifferentiated adipocytes show similar properties to bone marrow-derived mesenchymal stem cells. Stem Cells. 2012; 30: 965–974. 10.1002/stem.1067 22367678

[13] Obinata D, Matsumoto T, Ikado Y, Sakuma T, Kano K, Fukuda N, et al Transplantation of mature adipocyte-derived dedifferentiated fat (DFAT) cells improves urethral sphincter contractility in a rat model. Int J Urol. 2011; 18: 827–834. 10.1111/j.1442-2042.2011.02865.x 21991997

[14] Jumabay M, Matsumoto T, Yokoyama S, Kano K, Kusumi Y, Masuko T, et al Dedifferentiated fat cells convert to cardiomyocyte phenotype and repair infarcted cardiac tissue in rats. J Mol Cell Cardiol. 2009; 47: 565–575. 10.1016/j.yjmcc.2009.08.004 19686758

[15] Ohta Y, Takenaga M, Tokura Y, Hamaguchi A, Matsumoto T, Kano K, et al Mature adipocyte-derived cells, dedifferentiated fat cells (DFAT), promoted functional recovery from spinal cord injury-induced motor dysfunction in rats. Cell Transplant. 2008; 17: 877–886. 19069631 10.3727/096368908786576516

[16] Ono H, Oki Y, Bono H, Kano K. Gene expression profiling in multipotent DFAT cells derived from mature adipocytes. Biochem Biophys Res Commun. 2011; 407: 562–567. 10.1016/j.bbrc.2011.03.063 21419102

[17] von Heimburg D, Lemperle G, Dippe B, Kruger S. Free transplantation of fat autografts expanded by tissue expanders in rats. Br J Plast Surg. 1994; 47: 470–476. 7524986 10.1016/0007-1226(94)90029-9

[18] Cinti S, Zingaretti MC, Cancello R, Ceresi E, Ferrara P. Morphologic techniques for the study of brown adipose tissue and white adipose tissue. Methods Mol Biol. 2001; 155: 21–51. 11293073 10.1385/1-59259-231-7:021

[19] Livak KJ, Schmittgen TD. Analysis of relative gene expression data using real-time quantitative PCR and the 2(-Delta Delta C(T)) Method. Methods. 2001; 25: 402–408. 11846609 10.1006/meth.2001.1262

[20] Nishimura S, Manabe I, Nagasaki M, Hosoya Y, Yamashita H, Fujita H, et al Adipogenesis in obesity requires close interplay between differentiating adipocytes, stromal cells, and blood vessels. Diabetes. 2007; 56: 1517–1526. 17389330 10.2337/db06-1749

[21] Jopling C, Boue S, Izpisua BJ. Dedifferentiation, transdifferentiation and reprogramming: three routes to regeneration. Nat Rev Mol Cell Biol. 2011; 12: 79–89. 10.1038/nrm3043 21252997

[22] Spalding KL, Arner E, Westermark PO, Bernard S, Buchholz BA, Bergmann O, et al Dynamics of fat cell turnover in humans. Nature. 2008; 453: 783–787. 10.1038/nature06902 18454136

[23] Yoshimura K, Eto H, Kato H, Doi K, Aoi N. In vivo manipulation of stem cells for adipose tissue repair/reconstruction. Regen Med. 2011; 6: 33–41. 10.2217/rme.11.62 21999260

[24] Suga H, Eto H, Shigeura T, Inoue K, Aoi N, Kato H, et al IFATS collection: Fibroblast growth factor-2-induced hepatocyte growth factor secretion by adipose-derived stromal cells inhibits postinjury fibrogenesis through a c-Jun N-terminal kinase-dependent mechanism. Stem Cells. 2009; 27: 238–249. 10.1634/stemcells.2008-0261 18772314

[25] Dodson MV, Hausman GJ, Guan L, Du M, Jiang Z. Potential impact of mature adipocyte dedifferentiation in terms of cell numbers. Int J Stem Cells. 2011; 4: 76–78. 24298337 10.15283/ijsc.2011.4.1.76 PMC3840976

[26] Cristancho AG, Lazar MA. Forming functional fat: a growing understanding of adipocyte differentiation. Nat Rev Mol Cell Biol. 2011; 12: 722–734. 10.1038/nrm3198 21952300 PMC7171550

[27] Avram MM, Avram AS, James WD. Subcutaneous fat in normal and diseased states 3. Adipogenesis: from stem cell to fat cell. J Am Acad Dermatol. 2007; 56: 472–492. 17317490 10.1016/j.jaad.2006.06.022

[28] Fan Y, Bergmann A. Apoptosis-induced compensatory proliferation. The Cell is dead. Long live the Cell!. Trends Cell Biol. 2008; 18: 467–473. 10.1016/j.tcb.2008.08.001 18774295 PMC2705980

[29] Suga H, Eto H, Aoi N, Kato H, Araki J, Doi K, et al Adipose tissue remodeling under ischemia: death of adipocytes and activation of stem/progenitor cells. Plast Reconstr Surg. 2010; 126: 1911–1923. 10.1097/PRS.0b013e3181f4468b 21124131

[30] Knopf F, Hammond C, Chekuru A, Kurth T, Hans S, Weber CW, et al Bone regenerates via dedifferentiation of osteoblasts in the zebrafish fin. Dev Cell. 2011; 20: 713–724. 10.1016/j.devcel.2011.04.014 21571227

[31] Kragl M, Knapp D, Nacu E, Khattak S, Maden M, Epperlein HH, et al Cells keep a memory of their tissue origin during axolotl limb regeneration. Nature. 2009; 460: 60–65. 10.1038/nature08152 19571878

[32] Eguizabal C, Montserrat N, Veiga A, Izpisua BJ. Dedifferentiation, transdifferentiation, and reprogramming: future directions in regenerative medicine. Semin Reprod Med. 2013; 31: 82–94. 10.1055/s-0032-1331802 23329641

[33] Eto H, Kato H, Suga H, Aoi N, Doi K, Kuno S, et al The fate of adipocytes after nonvascularized fat grafting: evidence of early death and replacement of adipocytes. Plast Reconstr Surg. 2012; 129: 1081–1092. 10.1097/PRS.0b013e31824a2b19 22261562

[34] Gao Q, Zhao L, Song Z, Yang G. Expression pattern of embryonic stem cell markers in DFAT cells and ADSCs. Mol Biol Rep. 2012; 39: 5791–5804. 10.1007/s11033-011-1371-4 22237862

[35] Ullah M, Stich S, Notter M, Eucker J, Sittinger M, Ringe J. Transdifferentiation of mesenchymal stem cells-derived adipogenic-differentiated cells into osteogenic- or chondrogenic-differentiated cells proceeds via dedifferentiation and have a correlation with cell cycle arresting and driving genes. Differentiation. 2013; 85: 78–90. 10.1016/j.diff.2013.02.001 23644554

[36] Piccolo S. Embracing mechanical forces in cell biology. Differentiation. 2013; 86: 75–76. 10.1016/j.diff.2013.08.001 24054842

[37] Jones PA, Baylin SB. The epigenomics of cancer. Cell. 2007; 128: 683–692. 17320506 10.1016/j.cell.2007.01.029 PMC3894624

[38] Choi M, Small K, Levovitz C, Lee C, Fadl A, Karp NS. The volumetric analysis of fat graft survival in breast reconstruction. Plast Reconstr Surg. 2013; 131: 185–191. 10.1097/PRS.0b013e3182789b13 23076412

[39] von Heimburg D, Hemmrich K, Zachariah S, Staiger H, Pallua N. Oxygen consumption in undifferentiated versus differentiated adipogenic mesenchymal precursor cells. Respir Physiol Neurobiol. 2005; 146: 107–116. 15766899 10.1016/j.resp.2004.12.013

